# A Novel Serine Protease Secreted by Medicinal Maggots Enhances Plasminogen Activator-Induced Fibrinolysis

**DOI:** 10.1371/journal.pone.0092096

**Published:** 2014-03-19

**Authors:** Mariena J. A. van der Plas, Anders S. Andersen, Sheresma Nazir, Nico H. van Tilburg, Peter R. Oestergaard, Karen A. Krogfelt, Jaap T. van Dissel, Paul J. Hensbergen, Rogier M. Bertina, Peter H. Nibbering

**Affiliations:** 1 Department of Infectious Disease, Leiden University Medical Center, Leiden, The Netherlands; 2 Department of Surgery, Leiden University Medical Center, Leiden, The Netherlands; 3 Department of Microbiology and Infection Control, Statens Serum Institute, Copenhagen, Denmark; 4 Novozymes A/S, Bagsvaerd, Denmark; 5 Copenhagen Wound Healing Center, Bispebjerg Hospital, Copenhagen, Denmark; 6 Department of Thrombosis and Hemostasis, Leiden University Medical Center, Leiden, The Netherlands; 7 Center for Proteomics and Metabolomics, Leiden University Medical Center, Leiden, The Netherlands; Gentofte University Hospital, Denmark

## Abstract

Maggots of the blowfly *Lucilia sericata* are used for the treatment of chronic wounds. As haemostatic processes play an important role in wound healing, this study focused on the effects of maggot secretions on coagulation and fibrinolysis. The results showed that maggot secretions enhance plasminogen activator-induced formation of plasmin and fibrinolysis in a dose- and time-dependent manner. By contrast, coagulation was not affected by secretions. Biochemical studies indicated that a novel serine protease within secretions, designated Sericase, cleaved plasminogen to several fragments. Recombinant Sericase degraded plasminogen leading amongst others to the formation of the mini-plasminogen like fragment Val454-plasminogen. In addition, the presence of a non-proteolytic cofactor in secretions was discovered, which plays a role in the enhancement of plasminogen activator-induced fibrinolysis by Sericase. We conclude from our *in vitro* studies that the novel serine protease Sericase, with the aid of a non-proteolytic cofactor, enhances plasminogen activator-induced fibrinolysis.

## Introduction

Maggots of the green bottle blowfly *Lucilia sericata* are used for the treatment of many types of wounds including venous ulcers [1], traumatic and post-surgical wounds [2], osteomyelitis [3] and burns [4]. Maggots exert many effects that may be beneficial for wound healing. Earlier we reported maggot excretions/secretions to breakdown bacterial biofilms of *Staphylococcus aureus* and *Pseudomonas aeruginosa* [5], [6]. Furthermore, maggots ingest and subsequently kill bacteria in their digestive tract [7], although quorum sensing regulated virulence factors from *P. aeruginosa* may be toxic to maggots [8]. Moreover, we recently identified a potent antimicrobial peptide belonging to the defensin class, designated Lucifensin [9], and showed that it acts by targeting the bacterial cell wall precursor Lipid-II [10]. In addition to direct antibacterial effects, we showed that secretions inhibit pro-inflammatory responses of human neutrophils [11] and monocytes [12] without affecting the antimicrobial activities of these phagocytes. Moreover, maggot secretions skew the monocyte-macrophage differentiation away from a pro-inflammatory to a pro-angiogenic type [13]. Others reported accelerated fibroblast migration induced by maggot excretions/secretions [14], [15].

Despite these many activities, maggots are primarily known for debridement - removal of necrotic tissue and fibrin slough - of chronic wounds. At present, maggot debridement is explained to result from direct enzymatic activity of the excretions/secretions of maggots involving serine proteases, chemotrypsins, glycosidases, DNases and lipases [16], [17], [18], [19], [20], [21]. It has been reported that, after debridement has been accomplished by maggots, minor bleeding may occur [22] . Surprisingly, investigations on the effects of maggots on haemostatic processes have never been reported.

Haemostasis consists of 3 phases: primary haemostasis (platelet plug formation), coagulation and fibrinolysis. Coagulation refers to the formation of insoluble fibrin which stops haemorrhage and provides a provisional matrix essential for cell migration thereby aiding in the repair of damaged vessels and tissues [23], [24]. In a balanced wound healing process fibrin clots are broken down (during remodelling of the tissue) by the fibrinolytic system; plasminogen is converted by plasminogen activators (urokinase plasminogen activator [uPA] or tissue-type plasminogen activator [tPA]) to plasmin, which subsequently cuts the fibrin mesh by proteolytic degradation [24]. In chronic wounds, fibrin clots may be degraded by proteolytic enzymes derived from immune cells, like neutrophils and macrophages, and bacteria. These clots no longer support re-epithelisation and the formation of granulation tissue and therefore have to be removed [23], [25]. However, this cannot be accomplished by the wound components themselves as, for instance, fibrinolysis may be impaired in chronic wounds due to enhanced levels of the fibrinolysis inhibitor PAI-1 [26], [27]. This may result in the formation of necrotic tissue and fibrin slough which contain trapped leukocytes and are a rich source of nutrients for bacteria. If necrotic tissue and/or fibrin slough are left unattended, it is very difficult to keep the wound free of infection, to prevent excessive inflammatory responses and to ensure closure of the wound. Therefore, removal of impaired tissue by debridement or enhanced fibrinolysis is essential for healing of these wounds. Based upon the above considerations and clinical observations, the aim of this study was to investigate the effects of maggot secretions on coagulation and fibrinolysis.

## Materials and Methods

### Preparation of Maggot Secretions

Sterile second- and third-instar larvae of *Lucilia sericata* were a kind gift from BioMonde GmbH (Barsbüttel, Germany). Maggot secretions were collected as described [12]; concentrations of secretions are indicated in μg of protein/mL. In each assay, at least 3 different pools of maggot secretions were used.

### Coagulation Assays

Clot formation was measured after incubating 10 μL of secretions (final concentration 50 μg/mL) or H_2_O as a control with 90 μL of citrated plasma (prepared from blood collected in 1/10^th^ volume 3.2% sodium citrate) for 10 and 30 min at room temperature (RT). The activated partial thromboplastin time (APTT) was measured after addition of 100 μL of APTT reagent (Kordia Life Sciences, Leiden, The Netherlands) and 100 μL of 25 mM Ca^2+^ to the secretions/plasma mixture. The prothrombin time (PT) measurement was started by adding 200 μL of Thromborel S (Dade Behring BV, Leusden, The Netherlands) to the mixture whereas the thrombin time (TT-test) was initiated by 25 μL of Thrombin (100 U/ml; Enzyme Research Laboratories Inc, South Bend, IN, USA). The time needed for clot formation was measured at 37°C.

### Clot Lysis Assay

Clot lysis was measured by a turbidimetric method using 96-wells plates. Mixtures of 80 μL were made containing 75% citrated plasma, 7.5 U/mL of tissue-type plasminogen activator (tPA; kindly provided by TNO, Leiden, The Netherlands) and secretions (range 1.25–5 μg) or, as a control, H_2_O and transferred to the wells. Subsequently, 20 μL of a second mixture consisting of a 100 fold dilution of Innovin (Dade Behring) in TEA-buffer (containing 25 mM triethanolamine, 0.05% Tween and 50 mM NaCl) supplemented with 100 mM CaCl_2_ was added. Next, the plate was shaken for 30 s after which the absorbance (405 nm) was measured 60 times with intervals of 10 minutes at 31°C using a microplate reader (Tecan Group Ltd., Männedorf, Switzerland). The time needed to obtain 50% lysis of the clot (X50) was calculated. Results, being the average X50 of samples in duplicate, were normalized by dividing them by the X50 obtained in the absence of secretions (Ratio).

### Plasminogen Activation

The effect of secretions on the kinetics of fibrinolysis was investigated in a system of purified proteins using the chromogenic substrate for plasmin S2403. Mixtures were made containing tPA (25–600 U/mL), secretions (0.78–100 μg/mL) or, as a control H_2_O, Glu-plasminogen (Glu-Plg; 0.33–2.68 μM) and 1.5 mM S2403 (Chromogenix, Milano, Italy) and transferred to 96-wells plates. Next, the plates were shaken for 30 s after which the absorbance (405 nm) was measured 30 times with intervals of 20 s at 31°C using an ELISA reader. Subsequently, absorbance values were corrected for the absorbance in the absence of Glu-Plg and tPA (due to enzymatic activity present in secretions) at each time interval. The resulting values were plotted against time square and the slope of this line, which reflects the rate of plasmin production, was calculated for each sample. Next, the Δabsorbance/sec^2^ was converted into rates of plasmin production (nM) using purified human plasmin as a standard (Enzyme Research Laboratories, South Bend, IN, USA).

### Acid-urea Gel Electrophoresis

Acid-urea gels (AU-page; 10%) were prepared as described [28]. Glu-Plg (7 μM) in TEA buffer was incubated with tPA and/or secretions for the indicated time intervals at 31°C and mixed with sample buffer (9.5 M urea in 5% acetic acid) in an 1∶1 ratio. Next, samples were transferred to the slots and gels were run in 5% acetic acid for 90–120 min at 150 V using reversed polarity. Thereafter, gels were stained with PageBlue (Fermentas GmbH, St. Leon-Rot, Germany).

### SDS-gel Electrophoresis

Glu-Plg was incubated as described above. Samples were denatured at 85°C for 2 min in 1x Novex Tricine SDS sample buffer. Precast Novex 10–20% Tricine gels were run on the XCell SureLock Mini-Cell System in 1x Novex Tricine SDS Running Buffer for 90 min at 125 V. All Novex products were acquired from Life Technologies (Carlsbad, USA). Gels were stained with PageBlue.

### Gel Filtration

Maggot secretions were concentrated using a speedvac concentrator (Savant, Fullerton, CA, USA), filtered through a 0.22 μm filter and applied to a gel filtration column (HiPrep 16/60 Sephacryl S-100 High Resolution 1,000–10,000 Da; Amersham Biosciences, Buckinghamshire, UK). The column was subsequently run with a mixture of 20% (v/v) acetonitril and 0.1% (v/v) acetic acid in H_2_O for 60 min with a flow rate of 0.5 mL/min. Next, 96 fractions of 2 mL were collected, vacuum-dried, solubilised in H_2_O and tested in the clot lysis and plasminogen (Plg) activation assays.

### In Gel Digestion and Mass Spectrometry

Protein bands were excised from SDS-PAGE gels, reduced, alkylated and in-gel digested using trypsin (modified, sequencing grade, Promega) as previously described [29]. After digestion, peptides were collected using two rounds of extraction with 20 μL of 0.1% TFA and stored at −20°C prior to analysis by mass spectrometry.

For LC-MS analysis, samples were injected onto a nano-LC system (Ultimate, Dionex, Amsterdam, the Netherlands) equipped with a peptide trap column (Pepmap 100, 0,3 i.d.×1 mm) and an analytical column (Pepmap 100, 0.075 i.d.×150 mm, Dionex, Amsterdam, the Netherlands). The mobile phases consisted of (A) 0.04% formic acid/0.4% acetonitrile and (B) 0.04% formic acid/90% acetonitrile. A 45 min linear gradient from 0 to 60% B was applied at a flow rate of 0.2 μL/min. The outlet of the LC system was coupled to a HCTultra ion-trap mass spectrometer (Bruker Daltonics, Bremen, Germany) using a nano-electrospray ionisation source. Eluting peptides were analysed in the data dependent MS/MS mode over a 400–1600 *m/z* range. Mass spectra were evaluated using the DataAnalysis 3.1 software package (Bruker Daltonics, Bremen, Germany). MS/MS spectra were searched against the NCBInr database using the Mascot search algorithm (Matrix Science, London, UK), allowing mass tolerances of 1.5 Da for MS and 0.5 Da for MS/MS and one missed cleavage site. Carbamidomethylcysteine was taken as a fixed modification and oxidation of methionine as a variable modification.

### Identification of the Full-length Protease Sequence

A cDNA library was constructed using mRNA isolated from *Lucilia sericata* maggots and used for transposon assisted signal trapping as described [9], [30]. In short, double stranded cDNA was digested, size fractionated and fractions above 400 base-pairs (bp) were ligated into a signal trapping vector. Next, the ligation mixture was electroporated into *E. coli* DH10B (Invitrogen) and these bacteria were incubated and subsequently plated onto agar plates. Plasmid DNA was isolated from these *E. coli* colonies and used in a transposon tagging reaction. The resulting suspension was electroporated into *E. coli* and these bacteria were incubated and plated onto selective agar plates. Plasmid inserts were sequenced by GATC Biotech (Konstanz, Germany) using transposon specific primers and finally assembled into contigs, using the PhredPhrap package (http://www.phrap.org) [30], which were then annotated using the Pedant genome database [31], [32].

### Cloning and Recombinant Expression of Sericase

The Sericase coding sequence was amplified from cDNA using the Extensor high fidelity PCR system (AB Gene, Surrey, UK) and oligonucleotides Sericase-Forward (5′ACACAACTGGGGATCCACCATGAAAACCTTTATTGCTCTAAG-3′) and Sericase-Reverse (5′AGATCTCGAGAAGCTTATTTGTTGACAACACCAGAAACT-3′). The resulting 876 bp PCR product was double digested with BamHI and HindIII, and GFX purified. Next, the digested DNA fragments were cloned into the HindIII-BamHI double digested shuttle expression vector (pDAU109) as described in patent WO 2005/042735. Resulting plasmids, containing the full-length Sericase cDNA gene (including signal peptide), were cloned into *E. coli* DH10B cells, selected on LB supplemented with 100 mg/mL of ampicillin and verified by sequencing. Subsequently, plasmid DNA was GFX purified and transformed into the *Aspergillus oryzae* strain BECh2 (*amy*−, *alp*−, *NpI*−, *CPA*−, *KA*−), as described in patent WO 2005/042735 and Woeldike *et al* [33]. Next, ten transformants (actively growing and sporulating in the presence of acetamide) were re-isolated twice under selective conditions on Cove-N minimal media plates containing 10 mM acetamide and 1 M sucrose [34].

To test the expression and secretion of Sericase, transformants were grown in YPM media (1% (w/v) yeast extract, 2% (w/v) peptone and 2% (w/v) maltose) for 3 days at 30°C while shaking at 200 rpm. Next, supernatants were run on Novex NuPage 4–20% Tris glycine SDS gels as described above. Endopeptidase activity was verified by AZCL-Casein (Megazyme, Wicklow, Ireland) agar radial diffusion assays at pH 7.

### Purification of Sericase

A high yielding transformant was grown in YPM media for 3 days at 30°C while shaking at 200 rpm. Culture broth was filtered through a Whatman GF/F 0.7 μm glass fiber filter (Maidstone, UK) and subsequently through a 0.22 μm filter to remove host cells. Next, ammonium sulfate was added (with a final concentration of 1.6 M) and the solution was applied to a SOURCE Phenyl column (GE Healthcare, Buckinghamshire, UK) equilibrated with a buffer containing 20 mM succinic acid/NaOH and 1.6 M (NH_4_)_2_SO_4_ (pH 6). The column was washed with equilibration buffer and eluted with a linear gradient between the equilibration buffer and 20 mM succinic acid/NaOH (pH 6). Obtained fractions were tested for endopeptidase activity and the active fractions were concentrated using a 3 kDa cut-off Amicon Ultra centrifugal filter device (Millipore, Cork, Ireland) which was centrifuged at 4000×g for 45 min at 4°C. Subsequently, the concentrated fractions were applied to a Superdex 75 column (GE Healthcare) which was equilibrated and eluted with a buffer (pH 7) containing 100 mM H_3_BO_3_, 10 mM dimethylglutaric acid, 2 mM CaCl_2_ and 150 mM NaCl. Next, active fractions were pooled, the pH adjusted with acetic acid to pH 5 and diluted with deionized water to the same conductivity as 10 mM succinic acid/NaOH, 1 mM CaCl_2_, pH 5. This solution was applied to a SOURCE S column equilibrated and washed with a buffer containing 10 mM succinic acid/NaOH and 1 mM CaCl_2_ (pH 5). After elution with a linear NaCl gradient (0 to 0.15 M), fractions were tested for endopeptidase activity. Active fractions were inactivated by adding PMSF to a final concentration of 35 mM to the Novex Tris glycine SDS sample buffer and run on a 4–20% Tris-glycine SDS-PAGE as described above. Pure fractions (>95% pure) were pooled as the purified product. Sequence and MW of the purified protease was verified by Edman degradation, mass spectrometry and N-terminal sequencing.

### Identification of the Sericase Cleavage Site in Plasminogen

Glu-Plg was incubated with Sericase (100 μg/mL) for 90 min at 30°C in TEA buffer (final volume 60 μL) and inactivated by adding PMSF. The digest was run on a 4–20% Tris-glycine SDS-PAGE and bands representing the digest products were blotted onto a Problott PVDF membrane at 175 mA for 60 min, excised and subjected to N-terminal sequencing using a Procise 494 N-terminal sequencer from Applied Biosystems. Software used for the analysis was Procise PC v2.1. Simultaneously, a part of the digest was analysed by LC-MS on a Bruker MicroTOF using a BioBasic-4 column (5 μm, 300 Å, 2.1 mm ID×100 mm) from Thermo Scientific (Waltham, MA, USA). The mobile phase consisted of (A) 0.05% Trifluoroacetic acid and (B) 0.05% Trifluoroacetic acid/100% acetonitrile. A 20 min linear gradient from 6 to 100% was applied at a flow rate of 200 μL/min. Mass spectra were evaluated using the DataAnalysis 3.4 software package (Bruker Daltonics) and deconvoluted using MaxEnt. As sequence template for human Plg, we used the sequence GenBank: AAA60113.1 without aa1.19 comprising the signal peptide.

### Statistical Analysis

Statistical analyses were performed with a paired t-test using Graphpad Prism version 4.02. P<0.05 was considered significant.

## Results

### Effect of Secretions on Coagulation

To investigate whether maggot secretions interfered with the formation of fibrin clots, secretions were added to plasma. The effects of secretions on the intrinsic pathway, the extrinsic pathway and fibrin formation were assessed using the activated partial thromboplastin time (APTT), prothrombin time (PT) and thrombin time (TT), respectively. The results showed no effect of secretions (50 μg/mL) on coagulation (Table 1).

**Table 1:** Effect of secretions on coagulation.

	10 min		30 min	
Test	Control	Secretions	Control	Secretions
APTT	41.0±1.4	44.3±1.8	41.6±2.3	41.3±1.6
PT	25.3±0.2	22.4±0.3	24.0±0.3	22.2±0.2
TT	19.8±0.2	20.5±0.6	20.5±0.4	20.2±0.6

Citrate plasma and 50 μg of secretions/mL were incubated for 10 or 30 min at room temperature. Plasma clot formation, after addition of pathway specific substrates, was measured at 37°C. APTT, activated partial thromboplastin time; PT, prothrombin time; TT, thrombin time. The results, expressed in seconds, are means ± SEM of 6 experiments.

### Effect of Secretions on tPA-induced Fibrinolysis

**Figure 1:**
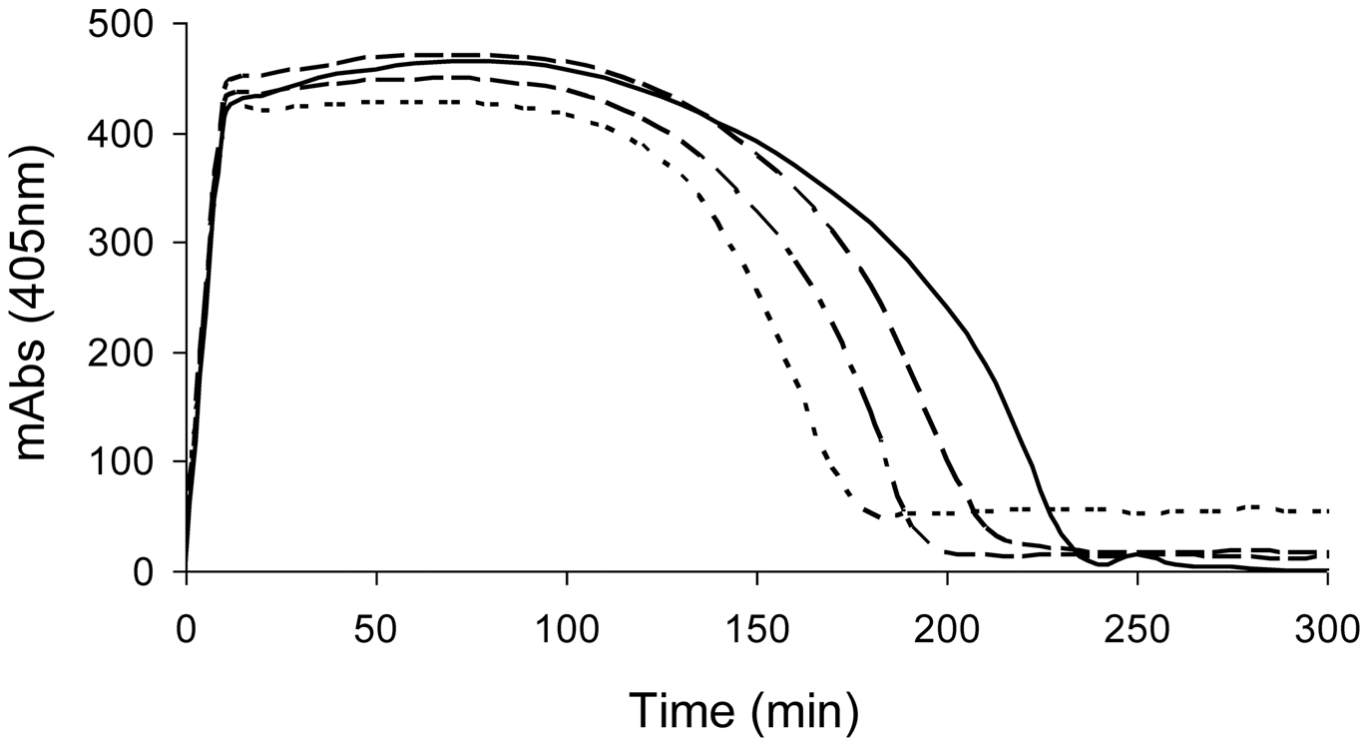
Effect of maggot secretions on the tPA-induced lysis of plasma clots (representative example). No secretions –; 12.5 μg of secretions/mL – – –; 25 μg of secretions/mL – – – –; 50 μg of secretions/mL – – –.

To investigate whether maggot secretions affect the lysis of plasma clots, a turbidimetric method was used. Plasma clots were formed in the presence of tPA and secretions, and the subsequent lysis of the clots was monitored at 405 nm. The results showed that secretions dose-dependently enhanced fibrinolysis, as the lysis time was decreased (Table 2; Figure 1). Secretions added to the wells 15 min after the fibrin clot was formed also reduced the lysis time, although less efficient, indicating that secretions are also effective against preformed clots. Similar results were obtained when using 30 U/mL of uPA instead of tPA (data not shown). Importantly, the addition of a Plg activator was essential as secretions alone did not induce clot lysis. Repeating the experiments with 50 μg of secretions/mL using plasma deficient in thrombin-activatable fibrinolysis inhibitor (TAFI) gave similar results as normal plasma (ratio of 0.79±0.009, p<0.005), whereas the lysis ratio of clots made from plasma derived from a patient with strongly reduced α2-antiplasmin activity was diminished (ratio of 0.90±0.020, p<0.005). As α2-antiplasmin inhibits the formation of plasmin from Glu-Plg much more efficiently than that of mini-Plg, these latter results suggest formation of mini-Plg-like fragments.

**Table 2:** Effect of secretions on the lysis time of fibrin clots.

Secretions (μg/mL)	Secretions added before clot formation	Secretions added after clot formation
0	1.00	1.00
12.5	0.94±0.03	0.96±0.09
25	0.87±0.02**	0.96±0.04
50	0.79±0.02**	0.92±0.03*
100	nd	0.86±0.06*
200	nd	0.81±0.04*

Clot formation was established by adding Innovin and Ca^2+^ to a mixture of citrated plasma and 6 U of tPA/ml. Maggot secretions were added directly to the plasma/tPA mixture or after the clot was formed. Breakdown of the clot was examined by measuring the optical density (405 nm) of the clot 60 times with 10 min intervals. Results, expressed as the ratio of the half lysis times (X50) compared with the control, are means ± SEM of 7–8 experiments. The X50 of the control was 207±2 min. Values are significantly (P<0.05)* or (p<0.005)** different compared to the control. nd: not done.

### Enhancement of tPA-induced Plasmin Formation by Secretions

**Figure 2:**
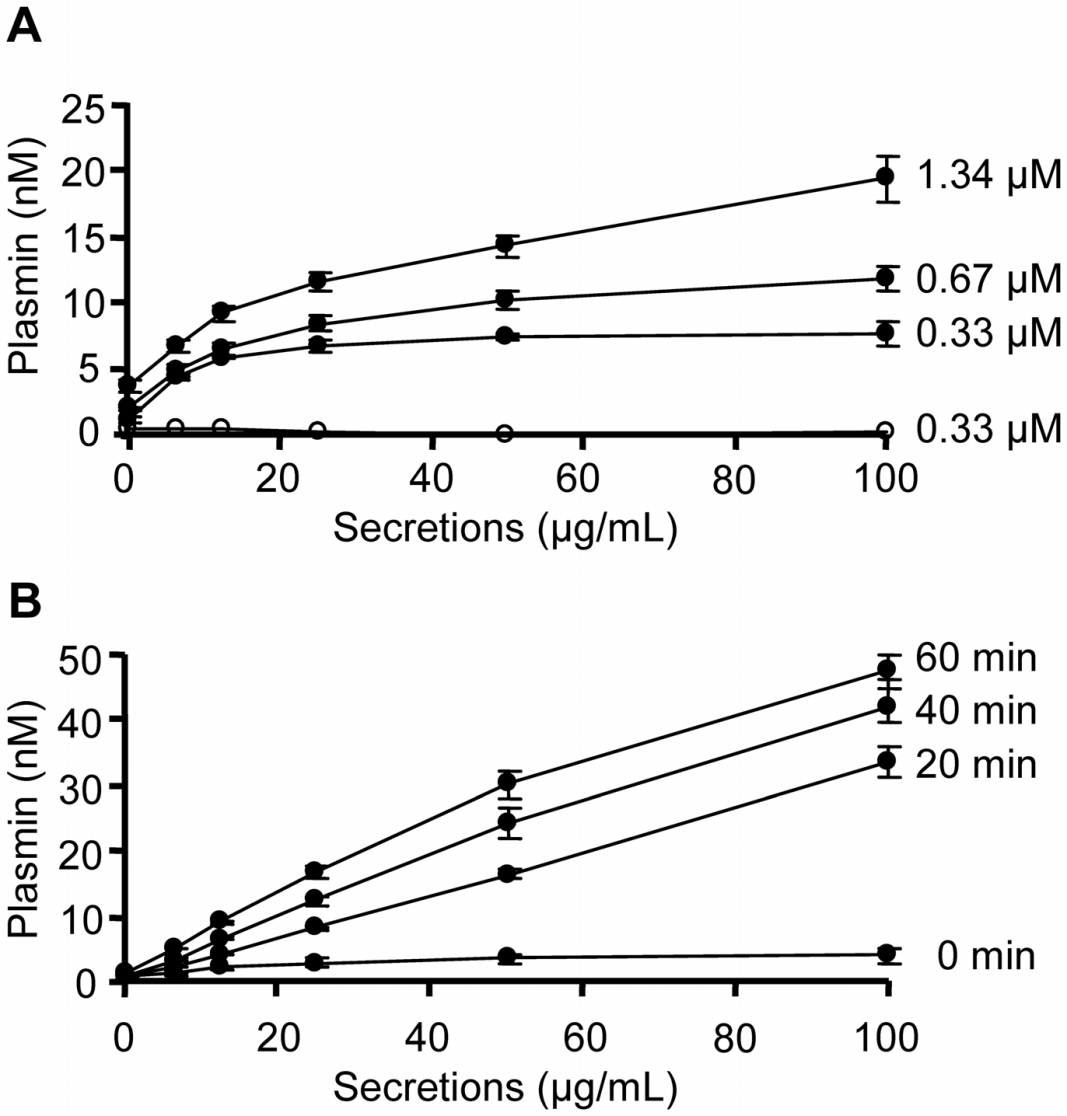
Effect of secretions on the tPA induced conversion of Glu-plasminogen. Secretions (1.56–100 μg/mL), tPA (50 U/mL) and Glu-Plg were mixed with substrate S2403 and substrate hydrolysis was measured at 405 nm. A) The effect of secretions on plasmin formation from various concentrations of Glu-Plg in the presence of tPA (closed circles) or no tPA (open circles). B) The effect of pre-incubating 0.33 μM Glu-Plg with secretions for various time intervals on tPA-induced plasmin formation. Results are means ± SEM of 3–5 experiments.

As the presence of a Plg activator was essential for secretions-stimulated fibrinolysis, we further studied the effect of secretions on tPA-induced Glu-Plg activation. To avoid interactions with plasmin inhibitors or other serum components, we used a system of purified proteins. The results showed that secretions enhanced the tPA (50 U/mL) mediated plasmin formation in the presence of Glu-Plg in a dose-dependent manner (Figure 2A). Moreover, increasing Glu-Plg concentrations enhanced the maximal effect of secretions, whereas increasing tPA concentrations had no additional effect (data not shown), indicating that secretions interact with Plg. Notably, secretions enhanced the tPA-induced plasmin formation from purified Lys-Plg as well (data not shown). In concordance with the effect on clot lysis, secretions alone were insufficient for plasmin formation. To investigate the stimulatory effect in more detail, secretions and Glu-Plg (0.33 μM) were incubated for various time intervals before addition of tPA (50 U/mL) and the plasmin substrate. The results showed that plasmin formation was dependent on the time that secretions and Glu-Plg were pre-incubated (Figure 2B). Incubating tPA with secretions before the addition of Glu-Plg and the plasmin substrate had virtually no effect on plasmin formation (data not shown).

### Effect of Secretions on Plasminogen

**Figure 3:**
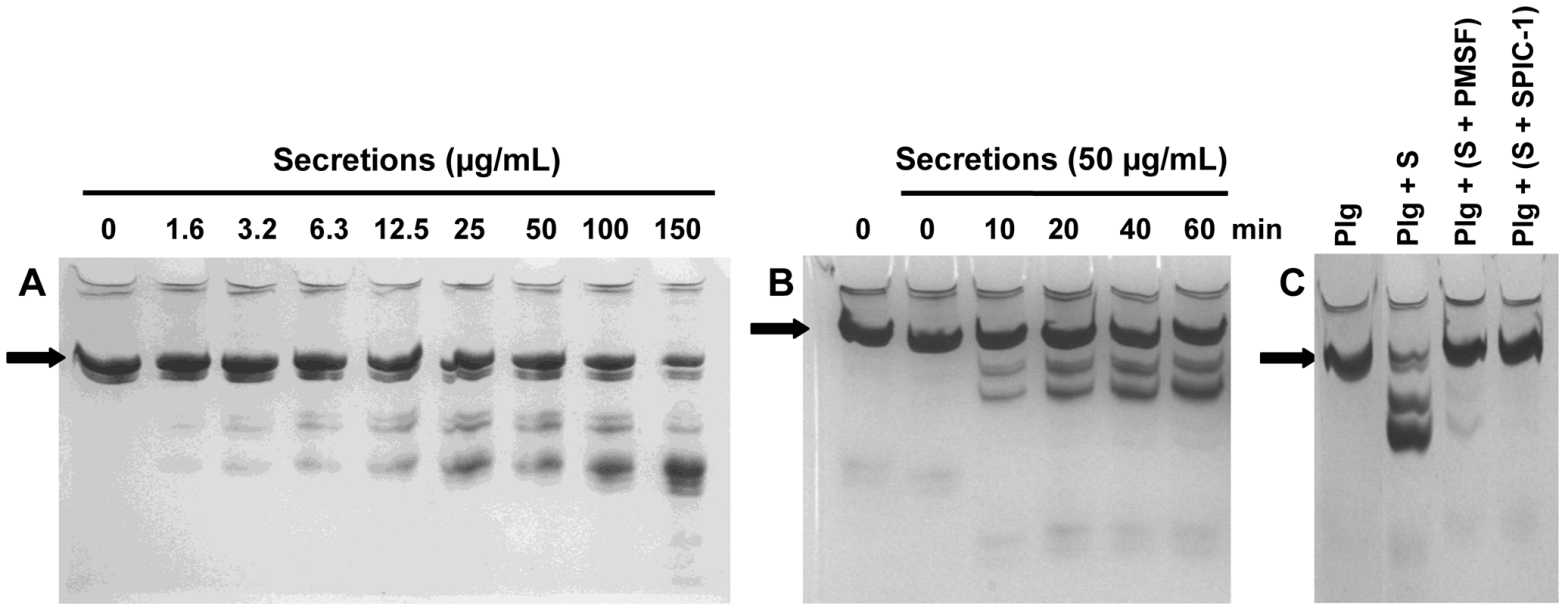
Effect of secretions on cleavage of Glu-plasminogen visualized with AU-page. Glu-Plg was incubated with A) various amounts of secretions for 1 h, or B) with 50 μg/mL of secretions for various time intervals. C) Glu-Plg was incubated for 1 h with a mixture of 50 μg of secretions(S)/mL pre-incubated for 24 h with the serine protease inhibitor PMSF or serine protease inhibitor cocktail 1 (SPIC-1). Black arrow: Glu-Plg.

Considering the results above, we investigated the possibility that secretions promote the proteolytic conversion of Plg into a derivative with a higher affinity for Plg activators. To be able to distinguish Glu-Plg from Lys-Plg and/or plasmin, we used acid-urea gel electrophoresis. The results showed that secretions cleave Glu-Plg in a dose- (Figure 3A) and time-dependent (Figure 3B) manner. Formation of Lys-Plg, a known derivative of Glu-Plg [35], and/or plasmin was not observed. Incubating secretions with 10 mM of PMSF or Serine Protease Inhibitor Cocktail Set I (Calbiochem, EMD Biosciences, Inc, La Jolla, Ca, USA) at 31°C completely abrogated the observed cleavage of Glu-Plg (Figure 3C), indicating that a serine protease within secretions is responsible for the observed effects.

### Isolation and Characterisation of the Active Component

**Figure 4:**
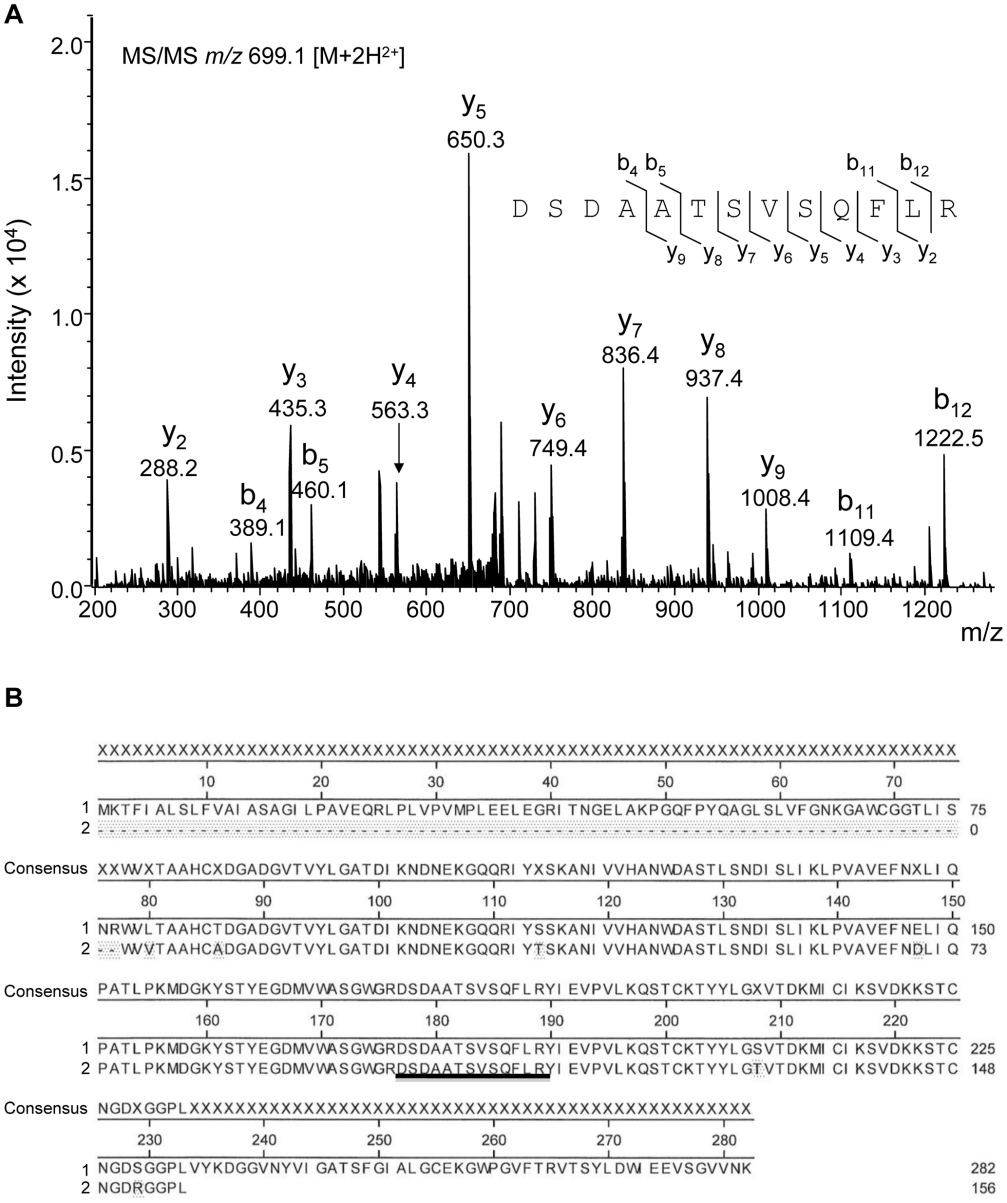
Characterisation of Sericase. Gel filtration fractions with fibrinolytic activity were run on a SDS-PAGE gel and protein bands were digested and analysed using LC-iontrap MS/MS (A). The full-length sequence of the active molecule, containing the 13 amino acid peptide, was identified using transposon assisted signal trapping. B1) Sericase including signal peptide (1.16), propeptide (17.40) and mature processed enzyme (41.282). B2) UNIPROTQ25236 partial *Lucilia cuprina* serine protease sequence. Shaded areas represent AA’s differing from Sericase. Alignment was performed using the clustal-W in DNAStar Meg-Align (Lasergene).

Next, we sought to characterise the active component from secretions. Using the clot lysis assay, we found that boiling secretions for two minutes or treating it with 0.1–1% of SDS or 6 M urea for 1 h at room temperature (RT) was sufficient to completely abrogate the pro-fibrinolytic effect of secretions (n = 3), indicating that the active component is a protein with a tertiary structure which is essential for its biological activity. Partial isolation by gel filtration resulted in three fractions (30 till 32) with fibrinolytic activity. These fractions were run on a SDS-PAGE gel and protein bands were digested with trypsin and analysed using LC-iontrap MS/MS. We found a 13 amino acid peptide (DSDAATSVSQFLR; Figure 4A) which showed full homology to a serine protease sequence from *Lucilia cuprina* (accession nr AAA17384/UNIPROTQ25236). Using transposon assisted signal trapping (TAST) we identified the full-length sequence of the serine protease from *L. sericata*, which we named Sericase (Figure 4B). The Sericase sequence was compiled from contigs recovered from a total of 87 clones out of 2000 sequenced indicating that the mRNA coding for this enzyme was an abundant transcript and therefore most likely highly expressed; both in the salivary glands and the digestive tract of the maggots (data not shown).

We recombinantly expressed Sericase in *Aspergillus oryzae* and studied its activity: characteristics of Sericase are given in Table 3.

**Table 3:** Characteristics of Sericase.

Theoretical MW 17.282	28707 Da
Theoretical MW 41.282	26069.51 Da
Observed MW 41.282	26069.11 Da
pH optimum	7.5
pH stability (>80% after 2 h)	4–10
Temperature optimum	45°C
N-terminal sequence	ITNGELAKPG

**Figure 5:**
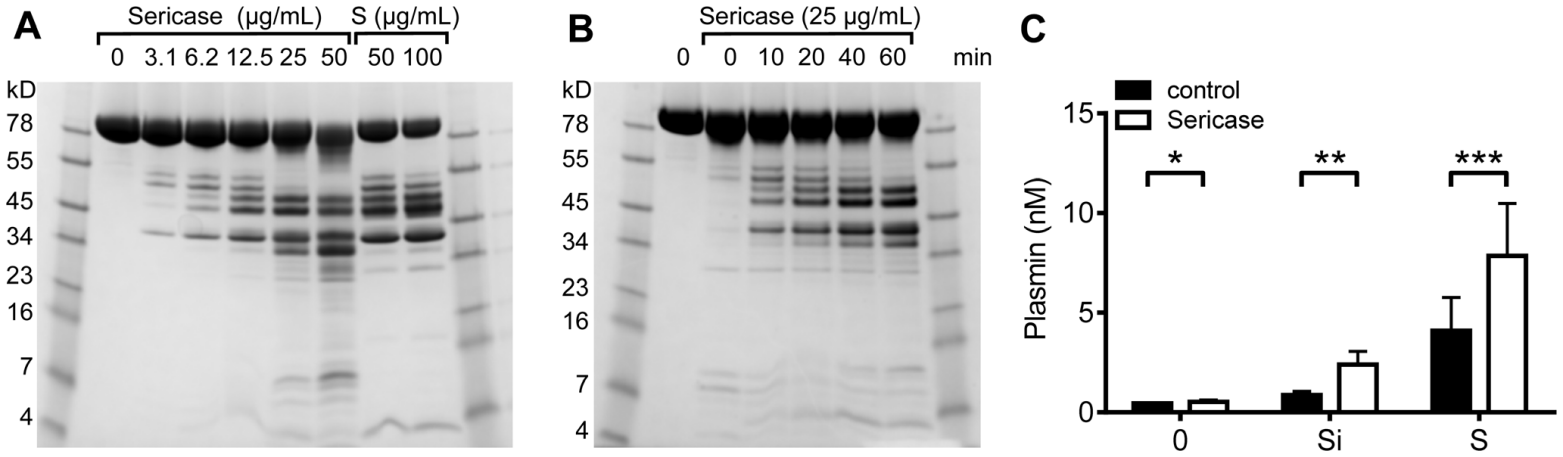
Effect of the Sericase on Glu-plasminogen cleavage and plasmin formation. A) Glu-Plg was incubated with various amounts of Sericase or secretions (S) for 1 h or B) incubated with 25 μg/mL of Sericase for various time intervals before running on 10–20% Tricine gels. The results are representative examples out of 3 experiments. C) 0.33 μM Glu-Plg was incubated for 20 min with 25 μg/mL of heat-inactivated secretions (Si) or regular secretions in the presence and absence of 25 μg/mL of Sericase before measuring plasmin formation. Results are means ± SEM of 6–10 experiments. Values are significantly (*p<0.05, p**<0.005 and p***<0.0005) different from those in the absence of Sericase.

Sericase dose- and time- dependently cleaved Glu-Plg (Figure 5A and 5B); the cleavage pattern of Sericase was similar to that of maggot secretions. However, Sericase was less active than secretions in enhancing the tPA-induced Glu-Plg activation. As activation of Plg by tPA is facilitated by denatured proteins from many sources [36], [37], [38], we investigated whether maggot secretions contain denatured proteins as well. For this purpose, 25 μg/mL of Sericase was incubated with 0.33 μM Glu-Plg in the absence or presence of 25 μg/mL of (heat-inactivated) secretions for 20 min at 31°C followed by the addition of 50 U/mL of tPA and substrate. The results showed that heat-inactivated secretions (and also normal secretions) considerably enhanced plasmin formation by Sericase (Figure 5C). Similar results were observed when Glu-Plg was incubated with Sericase alone for 20 min, followed by the addition of heat-inactivated secretions, tPA and substrate (data not shown). Incubation of Glu-Plg with heat-inactivated secretions for 20 min followed by the addition of Sericase, tPA and substrate led to a much smaller amount of plasmin formation. Together, these results indicate that within maggot secretions additional non-proteolytic co-factors are present that, after Sericase has cleaved Plg, contribute to the plasminogen activator-induced plasmin formation.

**Figure 6:**
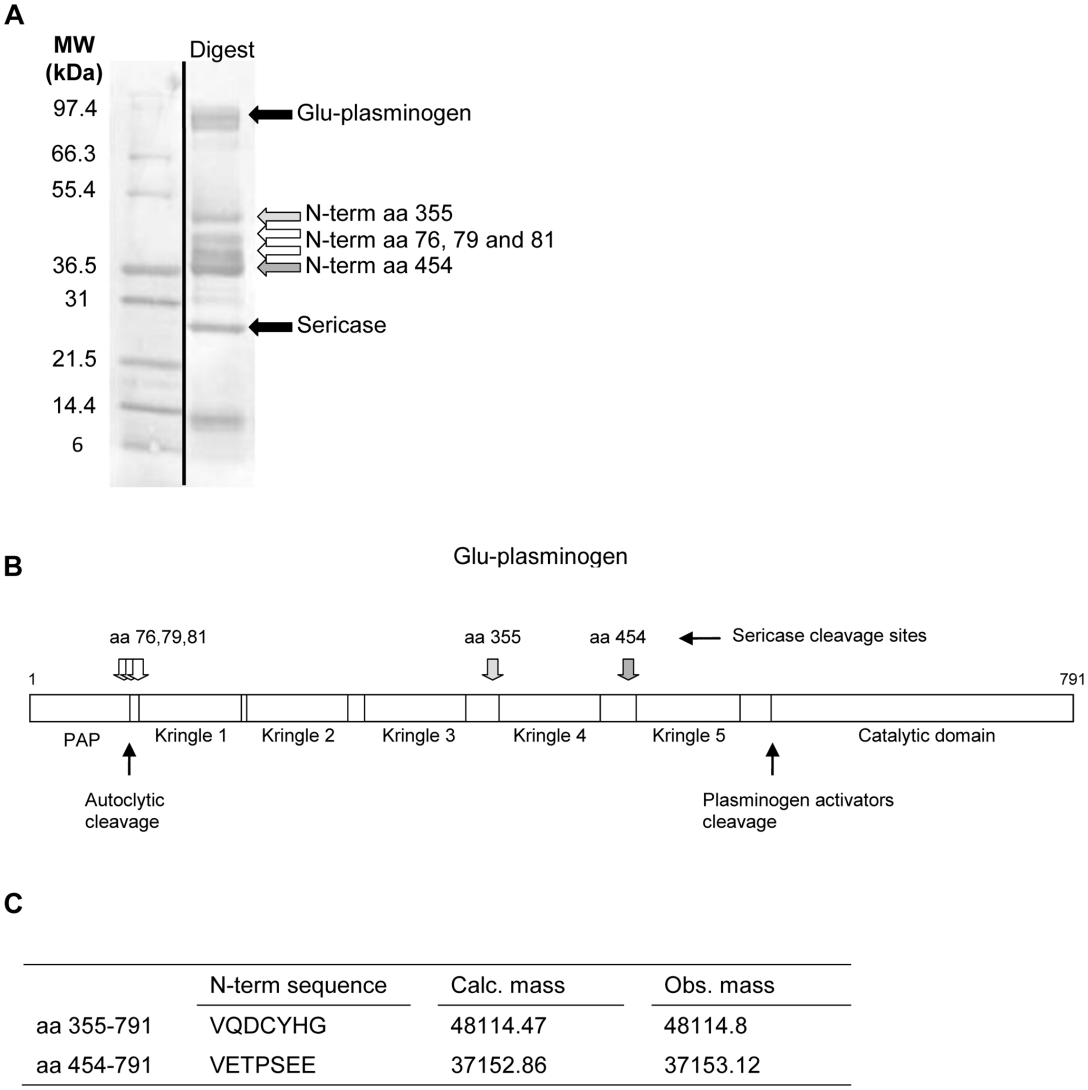
Identification of the cleavage products from plasminogen digested with Sericase. A) The digest run on a Tris Glycine 4–20% gel. The white and gray arrows show bands excised for N-terminal sequencing. The N-terminal cleavage sites are indicated as amino acid positions of Plg. B) Schematic overview of Plg and the cleavage sites for Sericase; PAP is preactivation peptide. C) Calculated and observed (by LC-MS) masses of the plasminogen derivates containing the catalytic domain.

To identify Sericase cleavage sites in Plg, we used N-terminal sequencing analysis of the major products and analysed the Glu-Plg digest with LC-MS (Figure 6A–C). We found that cleavage of Glu-Plg by Sericase resulted in two major cleavage products. The first product is a 48 kD intermediate comprising kringles 4 and 5 and the catalytic domain. This intermediate was cleaved further by Sericase to form a 37 kD molecule consisting of kringle 5 and the catalytic domain (Val454-Plg), which is very similar to mini-plasminogen (Val442-Plg) [39]. Additionally, Plg was further cleaved at amino acid positions 76, 79 and 81 leading to fragments of approximately 42 kD comprising kringles 1, 2 and 3.

## Discussion

The main conclusion from the current study is that the novel serine protease Sericase, secreted by medicinal maggots, enhances plasminogen activator-induced fibrinolysis by degrading plasminogen, thereby forming a mini-plasminogen-like fragment. This conclusion is based on the following observations. First of all, we found that tPA- and uPA-induced lysis of plasma clots was enhanced by secretions when added before as well as after the formation of the clots. Secretions did not induce lysis in the absence of plasminogen activators suggesting that it increases the affinity of Plg for its activators. In agreement, using a system of purified proteins we observed that secretions enhanced plasmin formation from Glu-Plg and Lys-Plg in the presence, but not in the absence, of tPA in a dose-dependent manner. Incubation of Glu-Plg and secretions resulted in cleavage of the protein into various fragments, and this digestion could be inhibited by serine protease inhibitors. We identified and recombinantly expressed a novel serine protease present within maggot secretions which enhanced the tPA-induced plasmin formation from Glu-Plg. Using LC-MS and N-terminal sequencing, we found that this enzyme, designated Sericase, cleaved Plg leading to the formation of two Plg derivates containing the catalytic domain; one of them (Val454-Plg) is comparable to mini-plasminogen (Val442-Plg) and likely to be responsible for the observed enhanced fibrinolysis. In this context, it has been reported that mini-Plg is more easily activated by plasminogen activators than Glu-Plg [40]. Moreover, mini-plasmin is only one-tenth as sensitive as plasmin to inhibition by alpha2-antiplasmin [41]. Together, this can explain the observed enhancement of fibrinolysis by the serine protease. In agreement, the effect of secretions on clot lysis was diminished when using plasma deficient in alpha2-antiplasmin. As inhibition of plasmin and mini-plasmin activity will be more equal in the absence of this inhibitor, these results further support the conclusion that the formed mini-plasminogen-like fragment is responsible for the observed effects. Finally, we found that (a) non-proteolytic co-factor(s) in maggot secretions facilitated the enhanced tPA-induced activation of plasminogen.

Another important finding from this study is that maggot secretions have no effect on coagulation at concentrations that clearly affect fibrinolysis. Interestingly, it is reported that bleeding may occur after debridement of wounds by maggots [22], although contradictory findings have been reported [42]. A possible explanation could be that the bleeding results from lysis of the clot before the underlying tissue has healed. However, in none of the patients treated with the contained form of maggot therapy (biobags) bleeding has been observed [43]. It is therefore unclear whether bleeding is the result of crawling of maggots [44] or of the amount of lysing components, as it is likely that a large portion of the maggot products stick to the biobags leading to a reduced concentration of active molecules in wounds.

What could be the clinical relevance of our findings? In a balanced wound healing process proteases are involved in enzymatic formation and degradation of the clot/provisional matrix which is essential for remodelling and repair of the tissue [45]. However, proteases such as elastase and matrix metalloproteases (MMP 1, 2, 8 and 9) in chronic wounds not only partially degrade clots and extracellular matrix but, due to their excessive production [23], [25], [46], also damage surrounding healthy tissue which leads to continuing fibrin deposition. As a consequence, clots/matrices in chronic wounds obtain an altered composition and structure, as compared to those in acute wounds [47], [48], no longer support re-epithelialisation and granulation tissue formation, and therefore have to be removed. However, elevated levels of pro-inflammatory mediators, like TNF-α and C5a, in chronic wounds may lead to enhanced production of the fibrinolysis inhibitor PAI-1 [49], [50] as is reported for obese and diabetic patients [26], [27]. PAI-1 binds to and inactivates uPA and tPA which results in impaired lysis of clots and fibrin cuffs [26], [51]. Additionally, enhanced levels of methylglyoxal found in diabetic patients result in a further decreased activation of plasminogen [52]. Similarly, it has been reported that serine protease inhibitors, like PAI-1, prevent plasminogen-induced fibroblast migration into fibrin clot provisional matrix [53]. In agreement, impaired wound healing observed in uPA/tPA double-deficient and plasminogen-deficient mice results from the diminished ability of wound edge cells to migrate through the provisional and/or extracellular matrix [54], [55]. Clearly, reduced plasmin formation and subsequent failure to form granulation tissue and remove partially degraded clots may promote formation of ulcers and/or (more) necrotic tissue and/or fibrin slough, which facilitates bacterial colonization and infection, and consequently pro-inflammatory responses. Sericase, secreted by medicinal maggots, plays a major role in enhancing the plasminogen activator-induced fibrinolysis by cleavage of Plg into a more easily activated form. This could tip the balance of suboptimal levels of Plg activators into sufficient concentrations to lyse clots as well as fibrin cuffs below the wound surface. These effects of secretions may explain their ability to effectively debride wounds of necrotic tissue and fibrin slough. Notably, debridement is presently explained to result from direct enzymatic activity of the excretions/secretions of the maggots, whereas our results showed that secretions do not degrade plasma clots in the absence of plasminogen activators. This is in contrast with a previous report [56] showing that maggot ES induced lysis of a fibrin matrix and various purified clot/matrix components. This discrepancy was not the result of differences in collecting the maggot products (data not shown) but likely the result of differences in the composition of the clots, as the fibrin matrix contained only fibrin, whereas in our studies clots were formed using plasma which contains a large variety of other proteins that can be incorporated in the clot thereby altering the structure. Hence, the composition of the clot may be an important factor for the activity of the enzymes within secretions.

In addition to debridement, maggot secretions enhance cAMP levels in leukocytes resulting in reduced production of pro-inflammatory cytokines [11], [12] which may lead to reduced PAI-1 levels thereby further enhancing fibrinolysis. In conclusion, these actions of maggots may actively promote healing of chronic wounds by preventing on-going inflammation and tissue destruction thereby protecting the fragile regenerating wound bed and hence contribute to the beneficial effects of maggots in diabetic foot ulcers and other chronic wounds found in clinical studies.
