# Correction: A Novel Serine Protease Secreted by Medicinal Maggots Enhances Plasminogen Activator-Induced Fibrinolysis

**DOI:** 10.1371/journal.pone.0101646

**Published:** 2014-06-24

**Authors:** The PLOS ONE Staff

Figure legend 1 is incorrect. The correct figure legend is:

**Figure 1. Effect of maggot secretions on the tPA-induced lysis of plasma clots (representative example).** No secretions —; 12.5 µg of secretions/mL — — —; 25 µg of secretions/mL – — – —; 50 µg of secretions/mL – – –.
